# Noninvasive assessment of myocardial work during left ventricular isovolumic relaxation in patients with diastolic dysfunction

**DOI:** 10.1186/s12872-023-03156-4

**Published:** 2023-03-10

**Authors:** Ying Guo, Xiang Wang, Chen-guang Yang, Xu-yang Meng, Yi Li, Chen-xi Xia, Tao Xu, Si-xian Weng, You Zhong, Rui-sheng Zhang, Fang Wang

**Affiliations:** Department of Cardiology, Beijing Hospital, National Center of Gerontology, Institute of Geriatric Medicine, Chinese Academy of Medical Sciences, No. 1 Dahua Road, Beijing, P.R. China

**Keywords:** Diastolic dysfunction, Isovolumic relaxation, Myocardial work, Myocardial work during isovolumic relaxation

## Abstract

**Background:**

This study aims to investigate the value of myocardial work (MW) parameters during the isovolumic relaxation (IVR) period in patients with left ventricular diastolic dysfunction (LVDD).

**Methods:**

This study prospectively recruited 448 patients with risks for LVDD and 95 healthy subjects. An additional 42 patients with invasive measurements of left ventricular (LV) diastolic function were prospectively included. The MW parameters during IVR were noninvasively measured using EchoPAC.

**Results:**

The total myocardial work during IVR (MW_IVR_), myocardial constructive work during IVR (MCW_IVR_), myocardial wasted work during IVR (MWW_IVR_), and myocardial work efficiency during IVR (MWE_IVR_) of these patients were 122.5 ± 60.1 mmHg%, 85.7 ± 47.8 mmHg%, 36.7 ± 30.6 mmHg%, and 69.4 ± 17.8%, respectively. The MW during IVR was significantly different between patients and healthy subjects. For patients, MWE_IVR_ and MCW_IVR_ were significantly correlated with the LV E/e’ ratio and left atrial volume index, MWE_IVR_ exhibited a significant correlation with the maximal rate of decrease in LV pressure (dp/dt per min) and tau, and the MWE_IVR_ corrected by IVRT also exhibited a significant correlation with tau.

**Conclusions:**

MW during IVR significantly changes in patients with risks for LVDD, and is correlated to LV conventional diastolic indices, including dp/dt min and tau. Noninvasive MW during IVR may be a promising tool to evaluate the LV diastolic function.

**Supplementary Information:**

The online version contains supplementary material available at 10.1186/s12872-023-03156-4.

## Background

Myocardial strain analysis has been validated as a reliable method for evaluating myocardial function. However, strain parameters are load dependent [1]. Myocardial work (MW) is emerging as an alternative and promising tool, because it includes both systolic blood pressure (BP) and strain, making it less afterload dependent [2]. MW can be considered as the improvement of myocardial strain [3], and has been demonstrated to be useful both in healthy subjects, and in patients with cardiovascular diseases [4–7].

The diastolic assessment of the left ventricle (LV) remains challenging [8]. In several studies, MW parameters have exhibited some correlations with traditional LV diastolic parameters, such as the septal and lateral tissue Doppler e’, average E/e’ ratio, and maximal left atrial volume index (LAVI) [9–11]. However, MW covers a time interval of both ventricular systole and isovolumic relaxation (IVR). Parameters correlated to myocardial performance during IVR, such as strain rate during IVR, have been shown to be useful for detecting early diastolic abnormalities, and associated with global diastolic dysfunction [12–14]. However, the MW during IVR has not been previously investigated. Furthermore, the role of MW parameters during the IVR period derived from MW has never been evaluated in healthy subjects and patients.

The present study aims to investigate the value of measuring MW during IVR in patients with left ventricular diastolic dysfunction (LVDD). The hypothesis of measuring MW during IVR is a promising approach to assess the LV diastolic function.

## Methods

### Study population

**Fig. 1:**
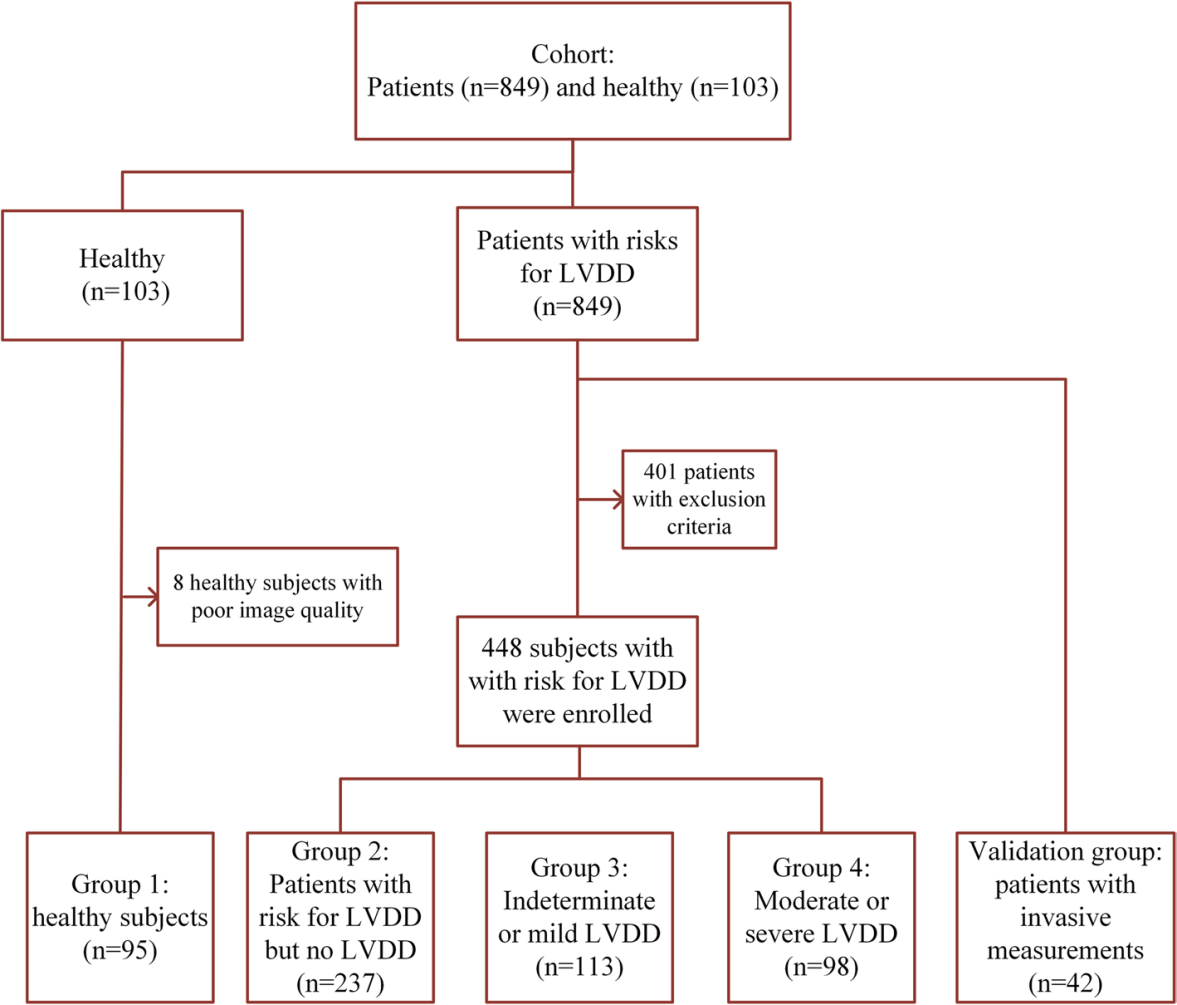
Flowchart for the selection of study participants. LVDD, left ventricular diastolic dysfunction

For the present study, 448 patients with risks for LVDD and 95 healthy subjects, who attended Beijing Hospital between October 2019 and August 2022, were consecutively included (Fig. 1).

The risks for LVDD included hypertension (systolic and diastolic BP ≥ 140/90 mmHg), hypercholesterolaemia (fasting plasma low-density lipoprotein [LDL] cholesterol ≥ 160 mg/dL), diabetes mellitus (fasting plasma glucose ≥ 126 mg/dl and glycated hemoglobin level ≥ 6.5%), and/or obesity (body mass index ≥ 30 kg/m^2^). The exclusion criteria were, as follows: (1) left ventricular ejection fraction (LVEF) of < 50%; (2) any pathological changes that could cause a pressure gradient between the aorta and left ventricle; (3) moderate or severe valvular heart disease; (4) arrhythmia, such as atrial fibrillation, supraventricular arrhythmias, left bundle branch block, etc.; (5) severe pulmonary, kidney, and/or liver disease; (6) the image quality for the speckle tracking analysis was poor.

Healthy subjects were defined, as follows: subjects who are free of any diseases and cardiovascular risk factors, such as hypertension, hyperlipemia, diabetes, obesity and coronary artery disease (CAD); subjects not receiving medications; subjects without abnormal findings in the routine transthoracic echocardiography, based on the guidelines of the American Society of Echocardiography (ASE) [15]. Healthy subjects were excluded when the image quality was poor in the speckle tracking analysis.

An additional 42 patients, who received LV catheterization due to suspected CAD, were prospectively recruited. The invasive measurements included the maximal rate of decrease in LV pressure (dp/dt min), time constant of LV isovolumic pressure decline (tau), and left ventricular end diastolic pressure (LVEDP).

The present study was approved by the institutional review board (IRB) (NCT03905200). All participants provided a signed informed consent.

### Echocardiography

Echocardiography was conducted by experienced sonographers using the Vivid E95 ultrasound system (GE Vingmed Ultrasound, Horten, Norway). Images in cine loop format were analyzed offline using the EchoPAC software (EchoPAC 204, GE Vingmed Ultrasound). All indices were measured according to ASE guidelines [15, 16]. Pulse Doppler imaging was used to measure the mitral valve peak early (E) and late (A) diastolic velocities, E/A ratio, and LV isovolumic relaxation time (IVRT). LVEF was calculated using the biplane Simpson’s method. LV global longitudinal strain (GLS) was defined as the average peak longitudinal strains obtained from three apical views [17]. Peak strain dispersion (PSD) was the standard deviation of the time-to-peak longitudinal strains for all segments [18].

According to the criteria of ASE [19], the cut-offs for abnormal LV diastolic performance were, as follows: (1) septal mitral annular e′ velocity of < 7 cm/s or lateral mitral annular e′ velocity of < 10 cm/s; (2) average E/e′ ratio of > 14; (3) LAVI of > 34 ml/m^2^; (4) peak tricuspid regurgitation velocity of > 2.8 m/s. The patients were diagnosed, as follows: LVDD, when > 50% of the indexes met the above criteria; indeterminate LVDD, when merely 50% of the criteria were positive; with risk of developing LVDD but not LVDD yet, when < 50% of the indexes met the above criteria [19]. For patients with LVDD, the severity of LVDD was defined according to the 2016 EACVI criteria [19, 20], as follows: mild, when E/A ≤ 0.8 and E ≤ 50 cm/s or ≥ 2 negative criteria (LAVI > 34 ml/m^2^, average E/e’ > 14, or TR > 2.8 m/s); moderate, when E/A ≤ 0.8 and E > 50 cm/s or 0.8 < E/A < 2 + ≥ 2 positive criteria (LAVI > 34 ml/m^2^, average E/e’ > 14, or TR > 2.8 m/s); severe, when E/A ≥ 2. Based on the above two criteria, the patients in the present study were categorized into three subgroups: patients with risks for LVDD but without LVDD (*n* = 237), patients with indeterminate or mild LVDD (*n* = 113), and patients with moderate or severe LVDD (*n* = 98). Among these patients, three patients met the criteria for mild LVDD, and seven patients met the criteria for severe LVDD.

### Conventional myocardial work parameters

In the EchoPAC software, the MW parameters were obtained through the pressure-strain loop (PSL) area module constructed from the curves for noninvasively estimated LV pressures and LV strains. The peak LV systolic pressure was assumed to be equal to the brachial cuff systolic BP measured during the echocardiographic study. This noninvasive method was validated by various research teams [1, 3, 4, 21, 22]. The myocardial work was calculated as the integral of power between mitral valve closure and mitral valve opening. The timings for the valvular events were defined on Doppler spectrums before entering the automated function imaging (AFI). The global work index (GWI) was defined as the total MW within the PSL area, from mitral valve closure to mitral valve opening. Global constructive work (GCW) was defined as the MW performed for shortening during ventricular systole and lengthening during IVR. Global wasted work (GWW) was defined as the MW performed for lengthening during ventricular systole and shortening during IVR. Global work efficiency (GWE) was calculated as the percentage of myocardial constructive work in the total MW (GCW / [GCW + GWW] × 100).

### MW parameters during the isovolumic relaxation period

The MW parameters for ventricular systole were derived by entering the timings of the mitral valve closure and aortic valve closure (defined from the Doppler trace at the aortic valve). Global systolic constructive work (GSCW) was defined as the MW during shortening in systole, and global systolic wasted work (GSWW) was defined as the MW during lengthening in systole. The MW parameters specific for IVR were calculated through deduction: MCW_IVR_ (myocardial constructive work during IVR, the myocardial work performed for lengthening during IVR) = GCW—GSCW; MWW_IVR_ (myocardial wasted work during IVR, the myocardial work performed for shortening during IVR) = GWW—GSWW. The total myocardial work during IVR (MW_IVR_) was obtained from the sum of MCW_IVR_ and MWW_IVR_. Myocardial work efficiency during IVR (MWE_IVR_) was calculated, as follows: MCW_IVR_ / (MCW_IVR_ + MWW_IVR_) × 100%. The MW_IVR_ parameters were normalized by dividing these by the corresponding IVRT.

### Invasive measurements

A total of 42 patients, who underwent LV catheterization for coronary angiography, were prospectively included. The invasive LV pressure was recorded. The LV dp/dt min, tau and LVEDP were averaged over 3–6 cardiac cycles. An LVEDP value of > 16 mmHg was defined as an elevated LV filling pressure [23]. The invasive values were measured by two researchers, who were blinded to the results of the MW measurements. All patients underwent coronary angiography with multiple projections. CAD was defined when the lumen was stenotic for more than 50% in one or more major epicardial coronary arteries [24].

### Statistical analysis

Continuous variables with normal distribution were expressed as mean ± standard deviation (SD), or median (interquartile range) when the normal distribution was not confirmed. The comparison of normally distributed variables between two groups was performed using independent-sample *t*-test. The comparison of non-normally distributed variables was performed using Mann–Whitney *U*-test. Comparisons among three or more groups of continuous variables were analyzed using analysis of variance (one-way ANOVA, non-normally distributed variables were log transformed). *X*^*2*^ or Fisher’s exact test was used for categorical data comparisons. Pearson’s correlation was used to test the association between MW parameters during IVR, and clinical or conventional echocardiographic variables, or dp/dt min, tau and LVEDP. The intra- and inter-observer variabilities of the MW parameters during IVR were assessed using intraclass correlation coefficients (ICCs). *P* < 0.05 was considered statistically significant. The statistical analysis was conducted using the SPSS 23.0 software.

## Results

### Comparison between patients in the different LVDD subgroups and normal subjects

The clinical and biochemical characteristics are presented in Table 1. The present study included 95 healthy patients, 237 patients with risk for LVDD but no confirmed LVDD, 113 patients with indeterminate or mild LVDD, and 98 patients with moderate or severe LVDD. The age, body mass index (BMI), and systolic BP were significantly lower in healthy subjects, when compared to the patients (*P* < 0.001). The levels of plasma brain natriuretic peptide (BNP) and uric acid were higher in the moderate or severe LVDD group, when compared to the levels in the other two patient groups (*P* < 0.005). There were no significant differences in the majority of the clinical and biochemical characteristics (BMI, diastolic BP, heart rate, HbA1c, fasting blood-glucose, LDL cholesterol, LPa and creatinine) among the patient groups (*P* > 0.05).

**Table 1:** Clinical characteristics of healthy subjects and patients

Variable	Healthy subjects (*n* = 95)	With risk for LVDD but no LVDD (*n* = 237)	Indeterminate or mild LVDD (*n* = 113)	Moderate or severe LVDD (*n* = 98)	*P-*value
Age, years	38.8 ± 8.6^†‡§^	62.6 ± 8.9^‡§^	66.5 ± 9.1^§^	69.1 ± 9.6	< 0.001
Men, *n* (%)	50 (52.6)^†^	164 (69.2)^§^	70 (61.9)	52 (53.1)	0.007
BMI, kg/m^2^	23.5 ± 3.0^†‡§^	25.8 ± 3.8	25.6 ± 3.1	26.4 ± 3.9	< 0.001
Systolic BP, mmHg	118.6 ± 13.8^†‡§^	130.3 ± 16.5^‡§^	135.4 ± 17.1	135.1 ± 15.8	< 0.001
Diastolic BP, mmHg	75.7 ± 10.5	76.0 ± 11.2	74.8 ± 10.7	75.0 ± 9.9	0.747
Heart rate, beats/min	68.7 ± 10.0^†‡§^	66.3 ± 9.7	65.4 ± 10.6	64.6 ± 9.4	0.024
**Cardiovascular risk factors**					
Hypertension, *n* (%)	0 (0)^†‡§^	160 (67.5)^‡^	93 (82.3)	75 (76.5)	< 0.001
Hypercholesterolemia, *n* (%)	0 (0)^†‡§^	168 (70.9)	85 (75.2)	67 (68.4)	< 0.001
DM, *n* (%)	0 (0)^†‡§^	102 (43.0)^§^	53 (46.9)	56 (57.1)	< 0.001
CAD, *n* (%)	0 (0)^†‡§^	131 (55.3)^‡^	49 (43.8)	44 (45.4)	< 0.001
**Biochemical indexes**					
BNP, pg/ml	-	27.4 (13.5–49.6)^§^	40.0 (21.4–77.8)^§^	76.9 (45.2–153.2)	< 0.001
HbA1c, %	-	6.6 ± 1.1	6.8 ± 1.1	6.8 ± 1.4	0.182
Fasting blood-glucose, mmol/L	-	6.1 ± 1.6^‡^	6.7 ± 2.3	6.4 ± 2.5	0.024
Uric acid, μmol/L	-	339.8 ± 77.5^§^	341.8 ± 98.9^§^	412.2 ± 446.1	0.021
LDL_C, mmol/L	-	2.2 ± 0.7	2.3 ± 0.8	2.1 ± 0.8	0.335
Homocysteine, μmol/L	-	11.9 ± 3.4^‡§^	14.0 ± 8.0	13.8 ± 7.8	0.005
LPa, mg/L	-	99 (43.5–192.5)	101 (41.3–269.8)	116 (17.5–261.5)	0.973
Creatinine, μmol/L	-	70.7 ± 14.5	72.5 ± 19.4	78.3 ± 50.8	0.086

*BNP* Brain natriuretic peptide, *CAD* Coronary artery disease, *BMI* Body mass index, *BP* Blood pressure, *DM* Diabetes mellitus, *LDL_C* Low-density lipoprotein cholesterol, *LVDD* Left ventricular diastolic dysfunction

^†^*P* < 0.05, compared to subjects with risks for LVDD but no LVDD

^‡^*P* < 0.05, compared to indeterminate or mild LVDD patients

^§^*P* < 0.05, compared to moderate or severe LVDD patients

The echocardiographic indices for LV systolic and diastolic performance were significantly different among the patient groups (Table 2). The PSD was higher in patients with intermediate/mild LVDD and moderate/severe LVDD. GWW was significantly lower, and GWE was significantly higher in healthy subjects, when compared to patients with risks for LVDD. There was no significant difference in GWW or GWE between patients with indeterminate or mild LVDD, and patients with moderate or severe LVDD.

**Table 2:** Echocardiographic data of healthy subjects and patients

Variable	Healthy subjects (*n* = 95)	With risk for LVDD but no LVDD (*n* = 237)	Indeterminate or mild LVDD (*n* = 113)	Moderate or severe LVDD (*n* = 98)	*P-*value
**Echocardiographic parameters**					
LVEF, %	65.2 ± 2.1^†‡§^	63.6 ± 4.1^‡§^	61.6 ± 6.8^§^	59.6 ± 8.5	< 0.001
IVRT, ms	64.3 ± 23.3^†‡§^	92.0 ± 31.9^‡§^	108.4 ± 36.3	100.7 ± 33.9	< 0.001
e’ Septal TDI, cm/s	0.13 ± 0.09^†‡§^	0.06 ± 0.02^‡§^	0.05 ± 0.01	0.05 ± 0.01	< 0.001
e’ Lateral TDI, cm/s	0.15 ± 0.03^†‡§^	0.09 ± 0.02^‡§^	0.06 ± 0.02	0.06 ± 0.02	< 0.001
Mitral E/e’ ratio	6.5 ± 1.5^†‡§^	9.7 ± 2.1^‡§^	15.0 ± 3.3^§^	18.4 ± 5.5	< 0.001
LAVI, ml/m^2^	18.5 ± 4.6^†‡§^	22.7 ± 6.5^‡§^	29.2 ± 7.0^§^	38.0 ± 7.0	< 0.001
LASr, %	37.4 ± 8.1^†‡§^	28.9 ± 7.4^‡§^	23.8 ± 6.0^§^	20.8 ± 6.9	< 0.001
PASP, mmHg	21.7 ± 3.9^†‡§^	26.2 ± 5.4^§^	26.4 ± 6.2^§^	30.6 ± 6.3	< 0.001
**GLS and MWs**					
GLS, %	-18.3 ± 2.5^†‡§^	-17.3 ± 2.7^‡§^	-16.5 ± 3.3	-16.3 ± 4.0	< 0.001
PSD, ms	45.7 ± 18.7^†‡§^	61.4 ± 32.5^‡§^	71.0 ± 32.1	71.1 ± 31.8	< 0.001
GWI, mmHg%	1,848.0 ± 302.1	1,830.7 ± 379.6	1,807.2 ± 475.2	1,810.6 ± 549.7	0.889
GCW, mmHg%	2,008.1 ± 294.8	2,058.0 ± 394.6	2,068.1 ± 498.2	2,020.9 ± 571.9	0.688
GWW, mmHg%	71.6 ± 38.3^†‡§^	105.5 ± 70.2^‡§^	141.8 ± 90.1	135.6 ± 79.4	< 0.001
GWE, %	95.7 ± 2.3^†‡§^	93.8 ± 3.8^‡§^	91.8 ± 5.5	91.5 ± 5.8	< 0.001
**MW**_**IVR**_** parameters**					
MW_IVR_, mmHg%	78.8 ± 41.9^†‡§^	116.7 ± 56.0^‡^	137.3 ± 65.7^§^	119.3 ± 60.5	< 0.001
MCW_IVR_, mmHg%	59.3 ± 34.1^†‡§^	85.7 ± 44.9^§^	95.5 ± 50.2^§^	74.5 ± 49.6	< 0.001
MWW_IVR_, mmHg%	19.5 ± 16.4^†‡§^	31.0 ± 29.3^‡§^	41.8 ± 33.3	44.8 ± 27.8	< 0.001
MWE_IVR_, %	74.9 ± 13.7^‡§^	73.5 ± 15.7^‡§^	69.3 ± 17.3^§^	59.4 ± 19.7	< 0.001

*GCW* Global constructive work, *GLS* Global longitudinal strain, *GWE* Global work efficiency, *GWI* Global work index, *GWW* Global wasted work, *IVR* Isovolumic relaxation, *IVRT* Isovolumic relaxation time, *LASr* Left atrial longitudinal strain during reservoir phase, *LAVI* Maximal left atrial volume index, *LVEF* Left ventricular ejection fraction, *LVDD* Left ventricular diastolic dysfunction, *MCW*_*IVR*_ Myocardial constructive work during IVR, *MW* Myocardial work, *MW*_*IVR*_ Total myocardial work during IVR, *MWE*_*IVR*_ Myocardial work efficiency during IVR, *MWW*_*IVR*_, Myocardial wasted work during IVR, *PASP* Pulmonary artery systolic pressure, *PSD* Peak strain dispersion, *TDI* Tissue doppler imaging

^†^*P* < 0.05, compared to subjects with risks for LVDD but no LVDD

^‡^*P* < 0.05, compared to indeterminate or mild LVDD patients

^§^*P* < 0.05, compared to moderate or severe LVDD patients

### Associations between myocardial work parameters during the isovolumic relaxation period, and the clinical and echocardiographic variables in healthy subjects (Supplementary table 1).

The values for MW_IVR_, MCW_IVR_, MWW_IVR_ and MWE_IVR_ in healthy subjects are presented in Table 2. MW_IVR_, MCW_IVR_ and MWW_IVR_ were significantly correlated with LV IVRT and systolic BP. Both MW_IVR_ and MCW_IVR_ were mildly correlated with the left atrial longitudinal strain during the conduit phase (LAScd). No significant correlations were found between the MW parameters during IVR, and age or heart rate.

### Associations between myocardial work parameters during the isovolumic relaxation period, and the clinical and echocardiographic variables in patients (Supplementary table 1)

**Fig. 2:**
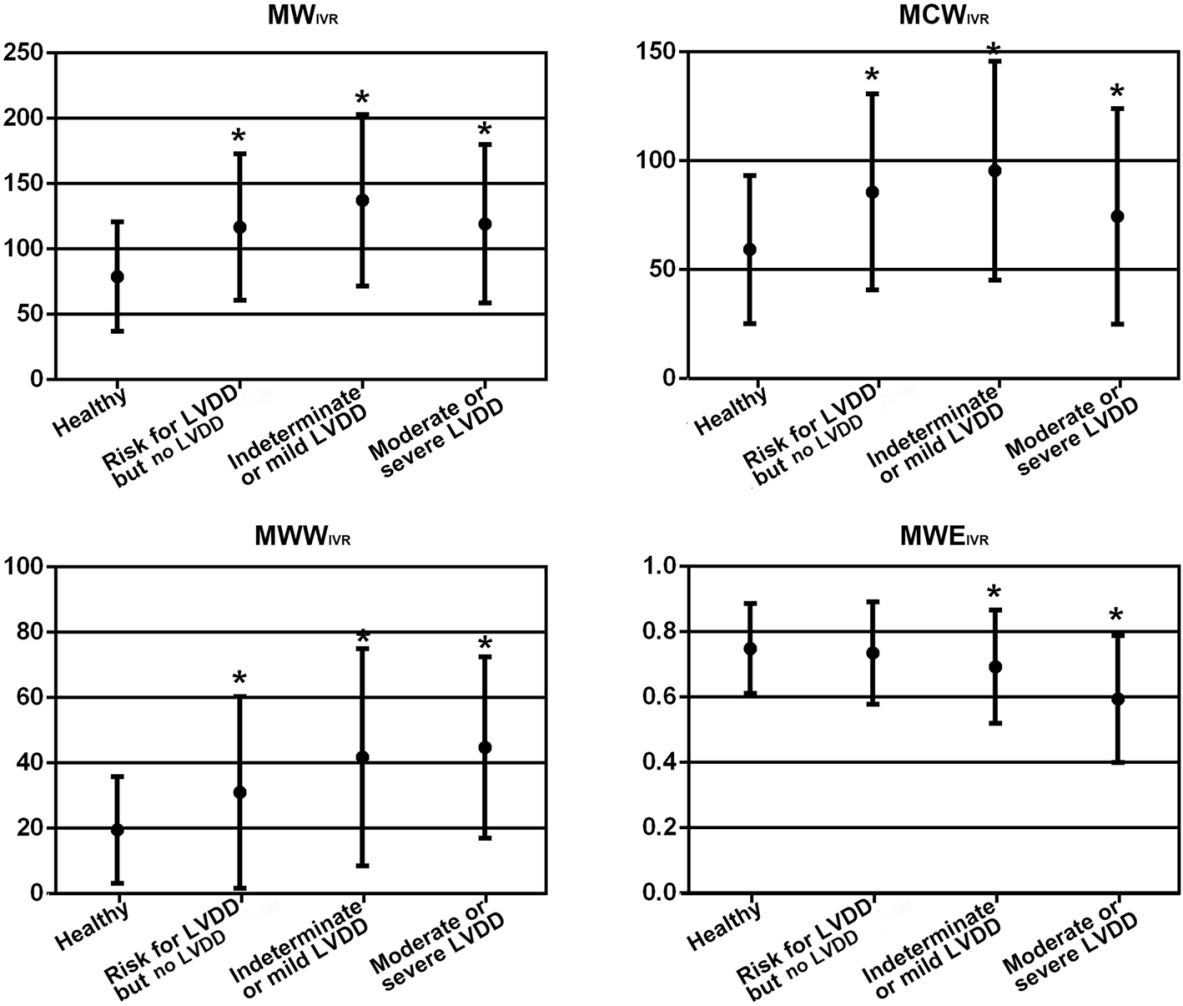
MW parameters during IVR, when compared across the different groups. IVR, isovolumic relaxation; LVDD, left ventricular diastolic dysfunction; MCW_IVR_, myocardial constructive work during IVR; MW_IVR_, total myocardial work during IVR; MWE_IVR_, myocardial work efficiency during IVR; MWW_IVR_, myocardial wasted work during IVR. ^*^P < 0.05, when compared to healthy subjects

MW_IVR_, MCW_IVR_ and MWW_IVR_ were significantly correlated with the IVRT. MWE_IVR_ exhibited a mild correlation with the IVRT (*r* = -0.121, *P* < 0.05). MW_IVR_ and MCW_IVR_ were associated with age and systolic BP. The MW parameters during IVR exhibited weak or no correlations with most of the left atrial strain parameters and serum biochemical indicators. No significant correlation was found between MW parameters during the IVR period and heart rate. MW_IVR_ and MCW_IVR_ were significantly higher in patients with risk for LVDD but no confirmed LVDD, when compared to healthy subjects. MW_IVR_ and MCW_IVR_ reached the maximum in patients with indeterminate or mild LVDD, and these declined in patients with moderate or severe LVDD (Table 2, Fig. 2). MWE_IVR_ exhibited a unidirectional change along with the severity of LVDD, with the lowest value in patients with moderate or severe LVDD (Table 2, Fig. 2). IVR ‘corrected’ MW_IVR_, MCW_IVR_ and MWW_IVR_ did not exhibit the dynamic pattern mentioned above when the diastolic dysfunction progressed. With the progress of the diastolic dysfunction, the IVR ‘corrected’ MWW_IVR_ gradually increased, while the IVR ‘corrected’ MWE_IVR_ gradually decreased (Supplementary table 2, Supplementary Fig. 1).

### Correlations among invasive measurements and myocardial work during the isovolumic relaxation period, myocardial work, and other diastolic parameters

**Fig. 3:**
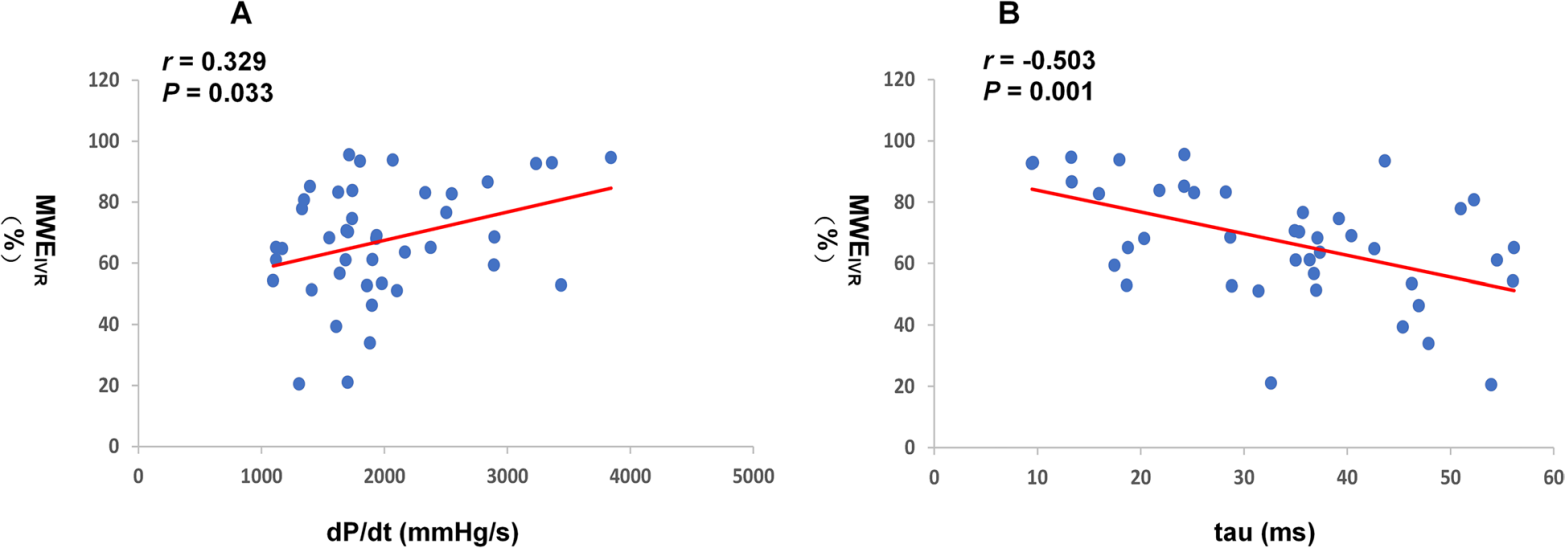
Correlations between dp/dt min and MWE_IVR_ (**A**), and correlations between tau and MWE_IVR_ (**B**); dp/dt min, the maximal rate of left ventricular pressure decrease; IVR, isovolumic relaxation; MWE_IVR_, myocardial work efficiency during IVR

The measurements obtained during catheterization are presented in Supplementary table 3. LVEDP was elevated in 26 patients (61.9%). The dp/dt min and tau were significantly correlated with MWE_IVR_ (*r* = 0.329*, P* = 0.033 and *r* = -0.503, *P* = 0.001, respectively; Table 3, Fig. 3). Normalized MWW_IVR_ and normalized MWE_IVR_ were significantly correlated with tau (*r* = 0.333*, P* = 0.031 and *r* = -0.316, *P* = 0.042, respectively; Supplementary table 4). The dp/dt min was significantly correlated with GWI, LASr and LASct. Tau was significantly correlated with GWE, GWI, GCW, LASr and LASct. LVEDP was significantly correlated with IVRT, e’ septal TDI, and LAVI (Supplementary table 4).

**Table 3:** Correlations between invasive measures of LV diastolic function and MW_IVR_ parameters (*n* = 42)

Variable	dp/dt min, mmHg/s		tau, ms		LVEDP, mmHg	
	**r**	***P***	**r**	***P***	**r**	***P***
MW_IVR_, mmHg%	-0.055	0.728	0.009	0.957	-0.216	0.170
MCW_IVR_, mmHg%	0.042	0.791	-0.162	0.305	-0.131	0.408
MWW_IVR_, mmHg%	-0.194	0.218	0.294	0.059	-0.266	0.089
MWE_IVR_, %	0.329^*^	0.033	-0.503^**^	0.001	-0.021	0.894

*dp/dt min* the maximal rate of left ventricular pressure decrease, *IVR* Isovolumic relaxation, *LVEDP* Left ventricular end diastolic pressure, *MCW*_*IVR*_ Myocardial constructive work during IVR, *MW*_*IVR*_ total myocardial work during IVR, *MWE*_*IVR*_ Myocardial work efficiency during IVR, *MWW*_*IVR*_ Myocardial wasted work during IVR. Normalized MW_IVR_ parameters, MW_IVR_ parameters corrected by IVRT

^*^P < 0.05

^**^P < 0.01

### Observer variabilities of myocardial work parameters during the isovolumic relaxation period

The intra- and inter-observer variabilities were measured in 30 randomly selected subjects. The ICC for intra-observer variability was 0.85 (95% CI: 0.71–0.93) for MW_IVR_ and 0.87 (95% CI: 0.75–0.94) for MWE_IVR_. The ICC for inter-observer variability was 0.85 (95% CI: 0.55–0.94) for MW_IVR_ and 0.85 (95% CI: 0.70–0.92) for MWE_IVR_.

## Discussion

MW parameters during IVR are novel echocardiographic parameters derived from MW. The present study was the first to report on MW parameters during the IVR period. The investigators identified novel MW parameters during IVR, which were correlated with the LV diastolic dysfunction measured by conventional echocardiography. In addition, it was identified that MW-derived indices, especially MWE_IVR,_ moderately correlated with the invasively measured dp/dt min and tau.

### Myocardial work parameters during isovolumic relaxation in healthy subjects

For the 95 healthy subjects, MW_IVR_, MCW_IVR_, MWW_IVR_ and MWE_IVR_ were not correlated with age or heart rate. These findings were different from the findings reported by Santoro et al*.* [25]. In the study conducted by Santoro et al*.*, 65% of the patients were < 49 years old, while in the present study, 85.3% of the patients were < 49 years old. Furthermore, a study revealed that the levels of GCW, GWW and GWE were stable until the age of 45 years old. Thereafter, there was an upward shift to further stable values of GCW, and a linear increase in GWW with the advance of age, resulting in lower GWE [9]. The difference in age may have contributed to the different findings between these studies. For the heart rate, the present results were consistent with the results reported by previously published studies [9, 26]. The MW during IVR increased with the increase in systolic BP. The impact of BP on MW indices was reported by a study [27, 28]. This impact appears to exist even within the physiological range of BP during the shorter period of the cardiac cycle, such as IVR. In addition, most of the MW_IVR_ parameters in the present study had very strong positive correlations with IVRT. This implies that the longer the IVR, the higher the total work of the myocardium.

Cardiac efficiency is the ratio between constructive work and total work (the sum of both constructive and wasted work). It was identified that in normal controls, MWE_IVR_ (74.9 ± 13.7%) was significantly lower than the GWE analyzed during systole and IVRT (95.7 ± 2.3%). This suggests that there is a higher proportion of wasted MW during the IVR interval, when compared to that in systole. The shortening, which is waisted and included in the calculation of wasted MW in IVR, is also called, post-systolic shortening [29]. Although widely deemed as a pathological sign, post-systolic shortening appeared to exist in healthy subjects in the present study. This was also observed by other studies [30, 31]. The quantification of myocardial work efficiency would help to further differentiate between patients and normal subjects.

### Indices for left ventricular diastolic dysfunction

Traditional LV diastolic function indices, such as septal and lateral e’, average E/e’ ratio and LAVI, are the recommended measurements for diastolic function analysis [19]. However, each index has its limitations [13, 20]. Therefore, the identification of optimal parameters for LV diastolic assessment remains as an ongoing pursuit, from both clinical and research perspectives. MW parameters during IVR may be good candidates. MW parameters during IVR were significantly correlated with the LV systolic and diastolic functional parameters in the present study. However, it was observed that similar to IVR [32], MW_IVR_ and MCW_IVR_ exhibited a dynamic pattern as the diastolic dysfunction progressed. This may limit its clinical application, since the MW values were lower in patients with moderate and severe LVDD, when compared to patients with indeterminate and mild LVDD. The significant correlation between these MW parameters and IVR duration was likely responsible for this pattern. As the left atrial pressure increased along with the diastolic dysfunction, the IVR was shortened after initially being prolonged [19]. After MW_IVR_ and MCW_IVR_ were normalized for IVRT time, these parameters no longer exhibited the dynamic pattern mentioned above as the diastolic dysfunction progressed. Among all the MW parameters, regardless of whether these were normalized or non-normalized, MWE_IVR_ was better and not impacted by the dynamic pattern. As shown in Fig. 2, MWE_IVR_ exhibited a unidirectional change as the diastolic dysfunction progressed, making it a good candidate for diagnosis, and stratifying the degree of diastolic dysfunction (9.2 ± 5.3%/s, 7.2 ± 3.6%/s, and 6.5 ± 4.5%/s, respectively, for subjects with risk for LVDD but no LVDD, indeterminate or mild LVDD patients, and moderate or severe LVDD patients, *P* < 0.05).

### Associations between myocardial work during isovolumic relaxation and invasive measurements

The noninvasive assessment of LV diastolic function remains challenging [19]. The correlations between some traditional parameters and invasive parameters were weak in the present study. The E/e’ ratio has been generally accepted for estimating the increase in LV filling pressure, and is included in the present guidelines and recommendations. However, the correlation between E/e’ and dP/dt min, tau, or LVEDP was poor in the present study (*r* = 0.084, *r* = 0.029 and 0.032, respectively). Similar results were also reported by other studies [33, 34]. Based on the present results, MWE_IVR_ is promising, since this was significantly associated with both tau (*r* = -0.503) and dp/dt min (*r* = 0.329). As the most established index to describe myocardial relaxation [35], the tau index measured during IVR was mostly correlated with MWE_IVR_. LVEDP is an important measurement for ventricular filling pressure, which is impacted by both myocardial relaxation and myocardial stiffness [36, 37]. This explains why there was no correlation between MW during IVR and LVEDP.

### Limitations

The present study had some limitations that should be mentioned. The average age of the healthy subjects was significantly lower, when compared to the patient groups. However, there was no significant correlation between age and MW parameters during IVR in healthy subjects. Hence, the impact of age on the MW parameters may not be significant. In the present study, echocardiography and cardiac catheterization were not simultaneously performed. Therefore, the pressure data during the IVR period was derived based on estimation, affecting the reliability of the MW parameters. Furthermore, the role of ventricular dyssynchrony was not comprehensively evaluated in the present study. Increased ventricular afterload would impair early relaxation, and induce wall dyssynchrony. The present results revealed that there was a mild correlation between PSD and MW_IVR_ parameters. However, further in-depth research on the dyssynchrony and contraction of myocardial fibers during IVRT is needed. Lastly, there were very few patients with mild and severe LVDD.

## Conclusions

MW during IVR progresses along with the severity of LVDD, and has some correlation with LV invasive diastolic indices, including dp/dt min and tau. Noninvasive MW during IVR may be a promising tool to evaluate the LV diastolic function.

## Supplementary Information

**Additional file 1:** **Supplementary Figure 1.** Normalized MW parameters during IVR, compared across the different groups. Legend: IVR, isovolumic relaxation; LVDD, left ventricular diastolic dysfunction; MCW_IVR_, myocardial constructive work during IVR; MW_IVR_, total myocardial work during IVR; MWE_IVR_, myocardial work efficiency during IVR; MWW_IVR_, myocardial wasted work during IVR. Normalized MW_IVR_ parameters, MW_IVR_ parameters corrected by IVRT. *P<0.05, compared to healthy subjects.

**Additional file 2: Supplementary table 1.** Associations between MW parameters during IVR, and clinical and echocardiographic variables in healthy subjects and patients. **Supplementary table 2.** Normalized MW_IVR_ parameters for healthy subjects and patients. **Supplementary table 3.** Baseline characteristics of patients with invasive measures of diastolic function (n=42). **Supplementary table 4.** Correlations between invasive measures of other parameters (n=42).

## Data Availability

The datasets used and/or analyzed in the study are available from the corresponding author on reasonable request.

## Abbreviations

ASE: American society of echocardiography
BMI: Body mass index
BNP: Brain natriuretic peptide
BP: Blood pressure
CAD: Coronary artery disease
CI: Confidence interval
DM: Diabetes mellitus
dP/dt min: Maximal rate of decrease in LV pressure
GCW: Global constructive work
GLS: Global longitudinal strain
GSCW: Global systolic constructive work
GSWW: Global systolic wasted work
GWE: Global work efficiency
GWI: Global work index
GWW: Global wasted work
ICCs: Intraclass correlation coefficients
IVR: Isovolumic relaxation
IVRT: Isovolumic relaxation time
LAScd: Left atrial longitudinal strain during conduit phase
LASct: Left atrial longitudinal strain during contraction phase
LASr: Left atrial longitudinal strain during reservoir phase
LAVI: Maximal left atrial volume index
LDL: Low-density lipoprotein
LDL_C: Low-density lipoprotein cholesterol
LV: Left ventricular
LVDD: Left ventricular diastolic dysfunction
LVEDD: Left ventricular end diastolic dimension
LVEDP: Left ventricular end diastolic pressure
LVEF: Left ventricular ejection fraction
MCW_IVR_: Myocardial constructive work during IVR
MW: Myocardial work
MWE_IVR_: Myocardial work efficiency during IVR
MW_IVR_: Myocardial total work during IVR
MWW_IVR_: Myocardial wasted work during IVR
PSD: Peak strain dispersion
PSL: Pressure-strain loop
TDI: Tissue doppler imaging
