# Supplementary material for: Noninvasive assessment of myocardial work during left ventricular isovolumic relaxation in patients with diastolic dysfunction
Source: BMC Cardiovasc Disord. 2023 Mar 10;23:129. doi: 10.1186/s12872-023-03156-4 (PMC9999647; doi:10.1186/s12872-023-03156-4)
Supplement: Supplementary file 1 — Additional file 1: Supplementary Figure 1. Normalized MW parameters during IVR, compared across the different groups. Legend: IVR, isovolumic relaxation; LVDD, left ventricular diastolic dysfunction; MCWIVR, myocardial constructive work during IVR; MWIVR, total myocardial work during IVR; MWEIVR, myocardial work efficiency during IVR; MWWIVR, myocardial wasted work during IVR. Normalized MWIVR parameters, MWIVR parameters corrected by IVRT. *P<0.05, compared to healthy subjects. [file 12872_2023_3156_MOESM1_ESM.docx]

**Supplementary Figure 1**

**
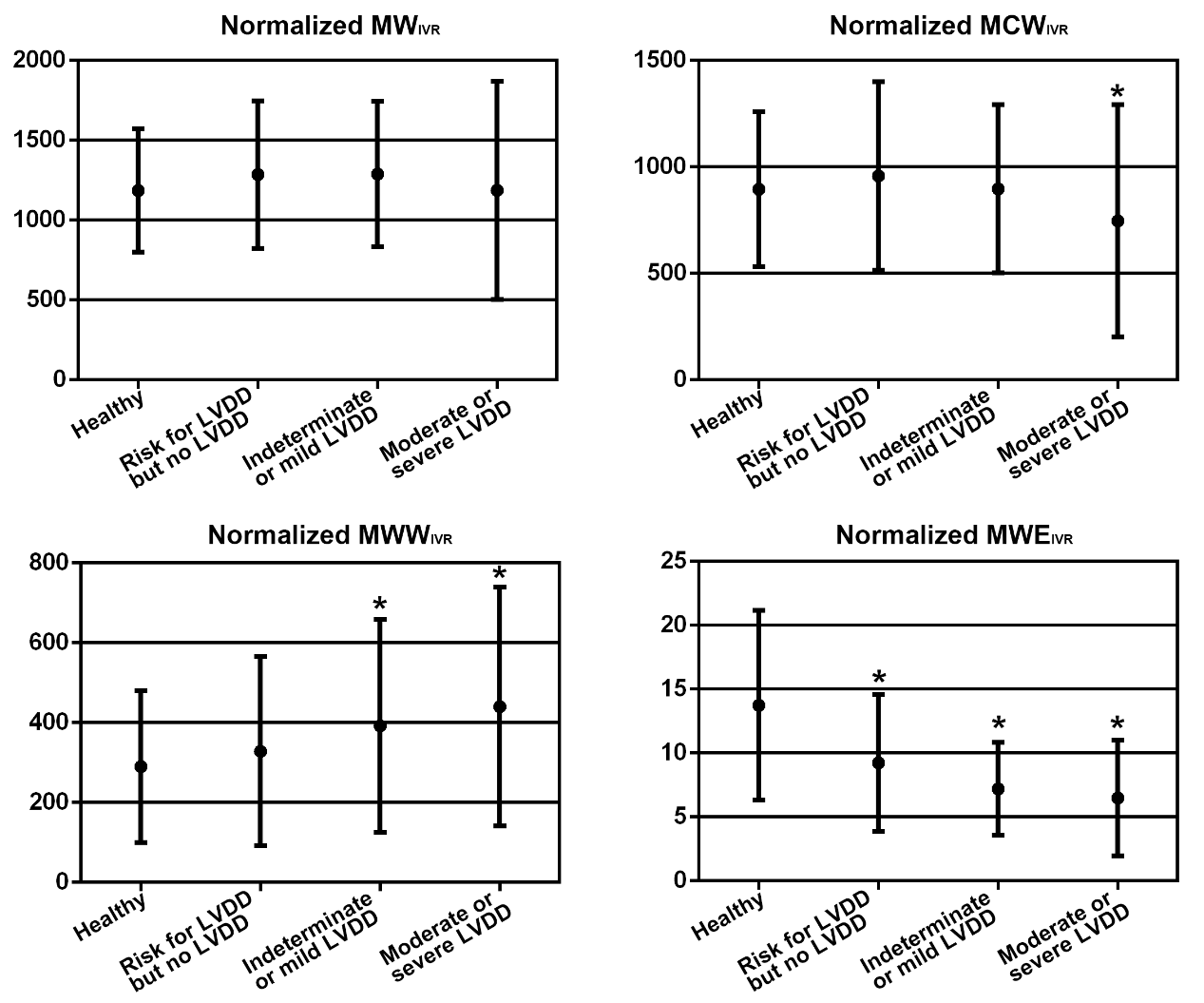
**

Figure Title:

Normalized MW parameters during IVR, compared across the different groups

Legend:

IVR, isovolumic relaxation; LVDD, left ventricular diastolic dysfunction; MCW_IVR_, myocardial constructive work during IVR; MW_IVR_, total myocardial work during IVR; MWE_IVR_, myocardial work efficiency during IVR; MWW_IVR_, myocardial wasted work during IVR. Normalized MW_IVR_ parameters, MW_IVR_ parameters corrected by IVRT. *P<0.05, compared to healthy subjects.
