# Supplementary material for: Noninvasive assessment of myocardial work during left ventricular isovolumic relaxation in patients with diastolic dysfunction
Source: BMC Cardiovasc Disord. 2023 Mar 10;23:129. doi: 10.1186/s12872-023-03156-4 (PMC9999647; doi:10.1186/s12872-023-03156-4)
Supplement: Supplementary file 2 — Additional file 2: Supplementary table 1. Associations between MW parameters during IVR, and clinical and echocardiographic variables in healthy subjects and patients. Supplementary table 2. Normalized MWIVR parameters for healthy subjects and patients. Supplementary table 3. Baseline characteristics of patients with invasive measures of diastolic function (n=42). Supplementary table 4. Correlations between invasive measures of other parameters (n=42). [file 12872_2023_3156_MOESM2_ESM.doc]

**Supplementary table 1. Associations between MW parameters during IVR, and clinical and echocardiographic variables in healthy subjects and patients**

| **Variable** | **MW_IVR_** | | **MCW_IVR_** | | **MWW_IVR_** | | **MWE_IVR_** | |
| --- | --- | --- | --- | --- | --- | --- | --- | --- |
|  | **r** | ***P*** | **r** | ***P*** | **r** | ***P*** | **r** | ***P*** |
| **Healthy subjects (*n*=95)** | | | | | | | | |
| Systolic BP, mmHg | 0.338 | 0.001^**^ | 0.289 | 0.005^**^ | 0.262 | 0.010^*^ | -0.083 | 0.425 |
| PSD, ms | 0.162 | 0.117 | 0.102 | 0.326 | 0.202 | 0.049^*^ | -0.190 | 0.066 |
| IVRT, ms | 0.825 | 0.000^**^ | 0.732 | 0.000^**^ | 0.587 | 0.000^**^ | -0.045 | 0.667 |
| Mitral E/e’ septal-lateral average ratio | -0.007 | 0.947 | 0.025 | 0.813 | -0.069 | 0.508 | 0.066 | 0.523 |
| LAVI, ml/m^2^ | 0.081 | 0.435 | 0.073 | 0.483 | 0.056 | 0.592 | 0.047 | 0.654 |
| LASr,% | -0.170 | 0.099 | -0.187 | 0.069 | -0.045 | 0.664 | -0.018 | 0.861 |
| LAScd,% | 0.262 | 0.010^*^ | 0.254 | 0.013^*^ | 0.142 | 0.171 | -0.020 | 0.851 |
| LASct,% | -0.066 | 0.524 | -0.022 | 0.834 | -0.124 | 0.232 | 0.060 | 0.563 |
| **Patients with LVDD risk factors (*n*=448)** | | | | | | | | |
| Age, years | 0.172 | 0.000^**^ | 0.169 | 0.000^**^ | 0.074 | 0.118 | 0.032 | 0.496 |
| Systolic BP, mmHg | 0.330 | 0.000^**^ | 0.288 | 0.000^**^ | 0.197 | 0.000^**^ | 0.016 | 0.733 |
| LVEF, % | -0.078 | 0.098 | 0.028 | 0.554 | -0.197 | 0.000^**^ | 0.191 | 0.000^**^ |
| GLS, % | -0.034 | 0.472 | -0.186 | 0.000^**^ | 0.222 | 0.000^**^ | -0.379 | 0.000^**^ |
| PSD, ms | -0.012 | 0.799 | -0.117 | 0.013^*^ | 0.159 | 0.001^**^ | -0.265 | 0.000^**^ |
| IVRT, ms | 0.592 | 0.000^**^ | 0.421 | 0.000^**^ | 0.506 | 0.000^**^ | -0.121 | 0.011^*^ |
| Mitral E/e’ septal-lateral average ratio | 0.040 | 0.393 | -0.064 | 0.175 | 0.179 | 0.000^**^ | -0.256 | 0.000^**^ |
| LAVI, ml/m^2^ | 0.040 | 0.288 | -0.024 | 0.617 | 0.136 | 0.004^**^ | -0.161 | 0.001^**^ |
| LASr,% | -0.132 | 0.005^**^ | -0.076 | 0.110 | -0.141 | 0.003^**^ | 0.144 | 0.002^**^ |
| LAScd,% | 0.168 | 0.000^**^ | 0.112 | 0.017^*^ | 0.155 | 0.001^**^ | -0.127 | 0.007^**^ |
| LASct,% | 0.032 | 0.493 | 0.002 | 0.967 | 0.061 | 0.200 | -0.095 | 0.044^*^ |
| BNP, pg/ml | -0.011 | 0.835 | -0.090 | 0.100 | 0.111 | 0.042^*^ | -0.197 | 0.000^**^ |
| Fasting blood-glucose, mmol/L | 0.048 | 0.323 | -0.010 | 0.845 | 0.108 | 0.027^*^ | -0.098 | 0.044^*^ |
| Uric acid, μmol/L | 0.019 | 0.690 | -0.057 | 0.242 | 0.125 | 0.010^*^ | -0.143 | 0.003^**^ |
| Homocysteine, μmol/L | 0.113 | 0.034^*^ | 0.108 | 0.042^*^ | 0.055 | 0.306 | -0.014 | 0.789 |
| LPa, mg/L | -0.221 | 0.022^*^ | -0.241 | 0.012^*^ | -0.054 | 0.579 | -0.112 | 0.251 |

^*^*P*<0.05, ^**^*P*<0.01; BNP, brain natriuretic peptide; BP, blood pressure; GLS, global longitudinal strain; IVR, isovolumic relaxation; IVRT, isovolumic relaxation time; LAScd, left atrial longitudinal strain during conduit phase; LASct, left atrial longitudinal strain during contraction phase; LASr, left atrial longitudinal strain during reservoir phase; LAVI, maximal left atrial volume index; LVDD, left ventricular diastolic dysfunction; LVEF, left ventricular ejection fraction; MCW_IVR_, myocardial constructive work during IVR; MW_IVR_, total myocardial work during IVR; MWE_IVR_, myocardial work efficiency during IVR; MWW_IVR_, myocardial wasted work during IVR; PSD, peak strain dispersion.

**Supplementary table 2. Normalized MW_IVR_** parameters for healthy subjects and patients

| **Variable** | **Healthy subjects**  **(*n*=95)** | **With risk for LVDD but no LVDD (*n*=237)** | **Indeterminate or mild LVDD (*n*=113)** | **Moderate or severe LVDD (*n*=98)** | ***P-*value** |
| --- | --- | --- | --- | --- | --- |
| Normalized MW_IVR_, mmHg%/s | 1,186.2 ± 387.4 | 1,286.2 ± 461.5 | 1,289.09 ± 455.3 | 1,187.0 ± 684.5 | 0.167 |
| Normalized MCW_IVR_, mmHg%/s | 896.0 ± 363.3^§^ | 957.8 ± 443.4^§^ | 896.9 ± 394.7^§^ | 746.5 ± 545.0 | 0.001 |
| Normalized MWW_IVR_, mmHg%/s | 290.1 ± 190.0^‡§^ | 328.4 ± 236.9^‡§^ | 392.2 ± 266.4 | 440.5 ± 299.2 | <0.001 |
| Normalized MWE_IVR_, %/s | 13.7 ± 7.4^†‡§^ | 9.2 ± 5.3^‡§^ | 7.2 ± 3.6 | 6.5 ± 4.5 | <0.001 |

^†^*P*<0.05, compared to subjects with risk for LVDD but no LVDD; ^‡^*P*<0.05, compared to indeterminate or mild LVDD patients; ^§^*P*<0.05, compared to moderate or severe LVDD patients. IVR, isovolumic relaxation; LVDD, left ventricular diastolic dysfunction; MCW_IVR_, myocardial constructive work during IVR; MW_IVR_, total myocardial work during IVR; MWE_IVR_, myocardial work efficiency during IVR; MWW_IVR_, myocardial wasted work during IVR; Normalized MW_IVR_ parameters, MW_IVR_ parameters corrected by IVRT.

**Supplementary table 3. Baseline characteristics of patients with invasive measures of diastolic function (*n***=42)

| **Variable** |  |
| --- | --- |
| Age, years | 62.1 ± 13.4 |
| Men, *n* (%) | 29.0 (69.0) |
| BMI, kg/m^2^ | 26.4 ± 4.6 |
| Systolic BP, mmHg | 131.7 ± 16.2 |
| Diastolic BP, mmHg | 77.2 ± 11.2 |
| Heart rate, beats/min | 70.9 ± 12.4 |
| LVEDP elevated, *n* (%) | 26.0 (61.9) |
| Echocardiographic data |  |
| LVEF, % | 58.6 ± 7.8 |
| IVRT, ms | 100.3 ± 32.3 |
| Mitral E/e’ septal-lateral average ratio | 12.9 ± 4.5 |
| LAVI, ml/m^2^ | 28.1 ± 8.1 |
| LASr, % | 22.8 ± 7.3 |
| GWI, mmHg% | 1,623.4 ± 559.6 |
| GCW, mmHg% | 1,903.1 ± 585.7 |
| GWW, mmHg% | 161.8 ± 107.9 |
| GWE, % | 89.7 ± 7.6 |
| MW_IVR_, mmHg% | 146.9 ± 74.9 |
| MCW_IVR_, mmHg% | 99.9 ± 57.0 |
| MWW_IVR_, mmHg% | 46.9 ± 33.5 |
| MWE_IVR_, % | 67.3 ± 18.9 |

BMI, body mass index; BP, blood pressure; GCW, global constructive work; GWE, global work efficiency; GWI, global work index; GWW, global wasted work; IVR, isovolumic relaxation; IVRT, isovolumic relaxation time; LASr, left atrial longitudinal strain during reservoir phase; LAVI, maximal left atrial volume index; LVEF, left ventricular ejection fraction; MCW_IVR_, myocardial constructive work during IVR; MW_IVR_, total myocardial work during IVR; MWE_IVR_, myocardial work efficiency during IVR; MWW_IVR_, myocardial wasted work during IVR.

**Supplementary table 4. Correlations between invasive measures of other parameters (*n*=42)**

| **Variable** | **dP/dt min, mmHg/s** | | **tau, ms** | | **LVEDP, mmHg** | |
| --- | --- | --- | --- | --- | --- | --- |
|  | **r** | ***P*** | **r** | ***P*** | **r** | ***P*** |
| Normalized MW_IVR_, mmHg%/s | -0.061 | 0.703 | -0.09 | 0.570 | -0.054 | 0.735 |
| Normalized MCW_IVR_, mmHg%/s | 0.067 | 0.676 | -0.261 | 0.095 | -0.018 | 0.912 |
| Normalized MWW_IVR_, mmHg%/s | -0.277 | 0.076 | 0.333^*^ | 0.031 | -0.098 | 0.536 |
| Normalized MWE_IVR_, %/s | 0.152 | 0.336 | -0.316^*^ | 0.042 | 0.046 | 0.773 |
| GWI, mmHg% | 0.309^*^ | 0.047 | -0.419^*^ | 0.006 | 0.077 | 0.628 |
| GCW, mmHg% | 0.294 | 0.059 | -0.425^*^ | 0.005 | 0.050 | 0.752 |
| GWW, mmHg% | -0.261 | 0.095 | 0.271 | 0.083 | -0.119 | 0.453 |
| GWE, % | 0.304 | 0.050 | -0.390^*^ | 0.011 | 0.060 | 0.704 |
| IVRT, ms | -0.013 | 0.936 | 0.135 | 0.394 | -0.325^*^ | 0.036 |
| e’ Septal TDI, cm/s | 0.174 | 0.271 | -0.268 | 0.086 | 0.323^*^ | 0.037 |
| e’ Lateral TDI, cm/s | 0.042 | 0.794 | -0.129 | 0.416 | 0.047 | 0.770 |
| Mitral E/e’ ratio | 0.084 | 0.597 | 0.029 | 0.858 | 0.032 | 0.843 |
| LAVI, ml/m^2^ | -0.188 | 0.232 | 0.094 | 0.554 | 0.341^*^ | 0.027 |
| LASr,% | 0.342^*^ | 0.027 | -0.327^*^ | 0.035 | -0.202 | 0.198 |
| LAScd,% | -0.130 | 0.411 | 0.081 | 0.609 | 0.020 | 0.899 |
| LASct,% | -0.338^*^ | 0.029 | 0.356^*^ | 0.021 | 0.227 | 0.148 |

^*^*P*<0.05, ^**^*P*<0.01; dP/dt min, the maximal rate of left ventricular pressure decrease; GCW, global constructive work; GWE, global work efficiency; GWI, global work index; GWW, global wasted work; IVRT, isovolumic relaxation time; LAScd, left atrial longitudinal strain during conduit phase; LASct, left atrial longitudinal strain during contraction phase; LASr, left atrial longitudinal strain during reservoir phase; LAVI, maximal left atrial volume index; LVEDP, left ventricular end diastolic pressure; MCW_IVR_, myocardial constructive work during IVR; MW_IVR_, total myocardial work during IVR; MWE_IVR_, myocardial work efficiency during IVR; MWW_IVR_, myocardial wasted work during IVR; Normalized MW_IVR_ parameters, MW_IVR_ parameters corrected by IVRT; TDI, tissue doppler imaging.
